# Individual and institutional influencing factors on completion rates in a medical education master’s program in Germany

**DOI:** 10.3205/zma001283

**Published:** 2019-11-15

**Authors:** Steffen Heide, Saskia V. Pante, Andreas Fleig, Andreas Möltner, Stefan Leis, Angelika Hiroko Fritz, Jana Jünger, Martin R. Fischer

**Affiliations:** 1 University of Halle-Wittenberg, Medical Faculty, Department of Forensic Medicine, Halle (Saale), Germany; 2 Heidelberg University, Medical Faculty, MME program, Heidelberg, Germany; 3 Heidelberg University, Medical Faculty, Center of Excellence for Assessment in Medicine, Heidelberg, Germany; 4 Paracelsus Medical University, University Clinic of Neurology, Salzburg, Austria; 5 University of Duisburg-Essen, Medical Faculty, Network Simulation Patient Program North Rhine-Westphalia, Essen, Germany; 6 Institute of Medical and Pharmaceutical Proficiency Assessment, Mainz, Germany; 7 LMU Munich, University Hospital, Institute for Medical Education, Munich, Germany

**Keywords:** Medical Education Master's Program, completion rates, influencing factors, outcomes research

## Abstract

**Background:** The increasing significance of didactic aspects in medical education has also led to the development of special postgraduate programs. Completion rates represent an important outcome criterion for these programs of study. Up to today, detailed studies on what factors influence these completion rates have been lacking.

**Methods: **Within the framework of outcomes research, a semi-structured online survey of students was conducted in the Master of Medical Education Germany program. Of the 90 items, 21 referred to the master's thesis that is required for graduation.

**Results: **157 out of 246 (63.8%) of students from classes 1-10 of the program (study period 2004-2014) participated in the survey. 109 participants had submitted a master's thesis, whereas 45 participants had not completed their studies by submitting a master's thesis. Influencing factors of successful completion were, among other things, little difficulty in choosing the topic, retention of the originally chosen topic, general support by the program administration in the modules and ensuring timely feedback from the advisor, and the provision of temporal, staff and financial resources by the home faculty. The failure to turn in the project report and a lengthy interruption of master thesi's work could be identified as critical parameters.

**Conclusion: **Taking into account these results can contribute to increasing completion rates in medical education graduate programs. Systematic outcomes research leads, moreover, to quality assurance. Such studies should be conducted in a standardized manner in the future, in order to facilitate comparisons between medical education programs.

## Zusammenfassung

**Hintergrund: **Die zunehmende Bedeutung didaktischer Aspekte in der Medizin hat auch zur Entwicklung besonderer Postgraduiertenprogramme geführt. Dabei bilden die Abschlussraten ein wichtiges Outcomekriterium für diese Studienprogramme. Bis heute fehlt es jedoch an detaillierten Studien dazu, welche Faktoren die Abschlussraten beeinflussen.

**Methoden:** Im Rahmen der Outcomeforschung wurde im Master of Medical Education-Programm Deutschland eine semistrukturierte Online-Umfrage unter Studierenden durchgeführt. Von den 90 Punkten bezogen sich 21 auf die Masterarbeit, die für den Abschluss erforderlich ist.

**Ergebnisse: **An der Umfrage nahmen 157 von 246 (63,8%) der Studierenden aus den Jahrgängen 1-10 (Studienzeitraum 2004-2014) teil. 109 Teilnehmer hatten eine Masterarbeit eingereicht, während 45 Teilnehmer ihr Studium noch nicht durch Abgabe einer Masterarbeit abgeschlossen hatten. Einflussfaktoren für einen erfolgreichen Abschluss waren unter anderem geringe Schwierigkeiten bei der Auswahl des Themas, Beibehaltung des ursprünglich gewählten Themas, allgemeine Unterstützung durch die Studienleitung in den Modulen sowie die Gewährleistung eines zeitnahen Feedbacks vom Betreuer und die Bereitstellung zeitlicher, personeller und finanzieller Ressourcen durch die Heimatfakultät. Die fehlende Einreichung der Projektarbeit und eine längerfristige Unterbrechung der Arbeit an der Masterarbeit konnten als kritische Parameter identifiziert werden.

**Schlussfolgerung: **Die Berücksichtigung dieser Ergebnisse kann zu höheren Abschlussraten bei Graduierten-Programmen in der Medizindidaktik führen. Eine systematische Ergebnisforschung führt zudem zu einer Qualitätssicherung. Solche Studien sollten zukünftig in standardisierter Form durchgeführt werden, um Vergleiche zwischen Medizindidaktikprogrammen zu erleichtern.

## 1. Background

The increased significance of didactic aspects in medical education in recent decades has also led to the development of special postgraduate programs [1], [2], [3], [4]. In 2013, already 121 study programs in medical education were offered internationally. The onset of this development in the German-speaking countries occurred with a certain delay. In 1996, a two-year postgraduate “Master of Medical Education” (MME) program, was established at the University of Bern in Switzerland [5]; in 2004, then followed the establishment of the German MME program. The Medical Faculty Association (MFT) is patron for the German MME course, which is administered and managed at Heidelberg University [6], [7]. In this two-year postgraduate part-time study program, participants take eight one-week classroom modules, each involving a follow-up assignment. Each module involves preliminary and follow-up tasks as well as proof of performance for the specific module; the total ECTS points for the modules is 36 points. In addition, in the first year, students prepare a project (9 ECTS points) on the improvement of instruction at their own faculties; and in the second year, they write a master's thesis (15 ECTS points) on an educational research project concerning a generalizable scientific problem It is not possible to successfully complete the program without submitting a master's thesis [7].

Besides commonalities, there are also considerable differences between the various medical education programs with respect to organization, structure and core contents [8], [9], [10], [11]. In 2016, the World Federation for Medical Education (WFME) first formulated standards in which criteria and mechanisms for evaluating these programs were formulated [12]. These standards pointed, among other things, to the fact that completion rates represent an important outcome criterion [12]. In the German MME program, the completion rates were around 72% for the first four classes. Up to date, however, detailed studies on what factors influence the completion rates in postgraduate medical education programs have been lacking [13]. It can be assumed that a series of different factors come into consideration [14]. In the case of sociodemographic factors, possible age- or sex-specific differences could play a role, for example. In addition, individual, external and program-immanent parameters can be taken into account. Individual factors are, in particular, the abilities or capacity of participants to fully meet the demands of the study program. External factors include, above all, infrastructure and supporting measures at the home faculties. Individual and external factors should, moreover, be considered in relation to the program-immanent characteristics. The hypothesis is put forward that successful completion of studies is significantly influenced, both negatively and positively, by several of such factors. The data from the German program is analysed, in order to provide an objective basis for this hypothesis.

## 2. Methods

The study of the influencing factors was combined with two other thematic complexes in a joint project on outcomes research concerning the MME program. These other thematic complexes concerned career and development of the graduates, as well as professional and personal inhibiting factors. A common catalogue of questions was conceived for a semi-structured online survey. The questionnaire mainly consists of closed questions and has some free text elements. Already validated and established scales, like KarMed, the Graduate Survey Cooperation Project (KOAB) questionnaire of the International Centre for Higher Education Research (INCHER) Kassel , and the E-PROM project questionnaire (funded by the German Federal Ministry of Education and Research-BMBF), were partially drawn upon here [15], [16], [17]. 

In a multi-stage process, the questionnaire was discussed, modified and tested by six current students and graduates of the program. The final version of the questionnaire comprised 90 individual questions, of which 35 items dealt with sociodemographic and professional information, 21 items with the MME master's thesis, 11 items with professional and personal inhibiting factors, and 23 items with career and development opportunities of the MME graduates (see supplemental data). 35 questions from the general part and 21 questions from the section on the master's thesis were used for the present study. The study was approved by the ethics committee of the Medical Faculty of Heidelberg University. 

246 former students from classes 1-10 (2004/05 to 2013/14 from the Heidelberg University) were invited by e-mail to participate in the study; the study was conducted using the “LimeSurvey” survey tool and was open for a period of six weeks (18/04-29/05/2016). Data entry took place using pseudonyms and was monitored by the program's administrative office. In addition, with the consent of the participants, internal comparative data was gathered for cohorts 1-10 and used for purposes of validation. 

Statistical analysis of the collected data was conducted using the free R environment (Version 3.4.3). Besides a descriptive analysis, this comprises an assessment of potential influencing factors on the completion of studies in form of the submission of the master's thesis. Since we do not only have the information about ‘submitted / not submitted’ but also on the individual submission dates, our analysis accounts for these temporal aspects. Therefore, we apply time-to-event analysis using log-rank tests and Kaplan-Meier curves. Both are common techniques used to identify differences in events – in our case submission of the master thesis – over the observation period among groups [18], [19]. Due to the limited number of observations and the data structure of missing information [20], further multivariate analysis techniques – like, for example, Cox regression [21], [22] – were not applicable. 

## 3. Results

### 3.1 Participation rate and time until submission of the master’s thesis 

**Figure 1:**
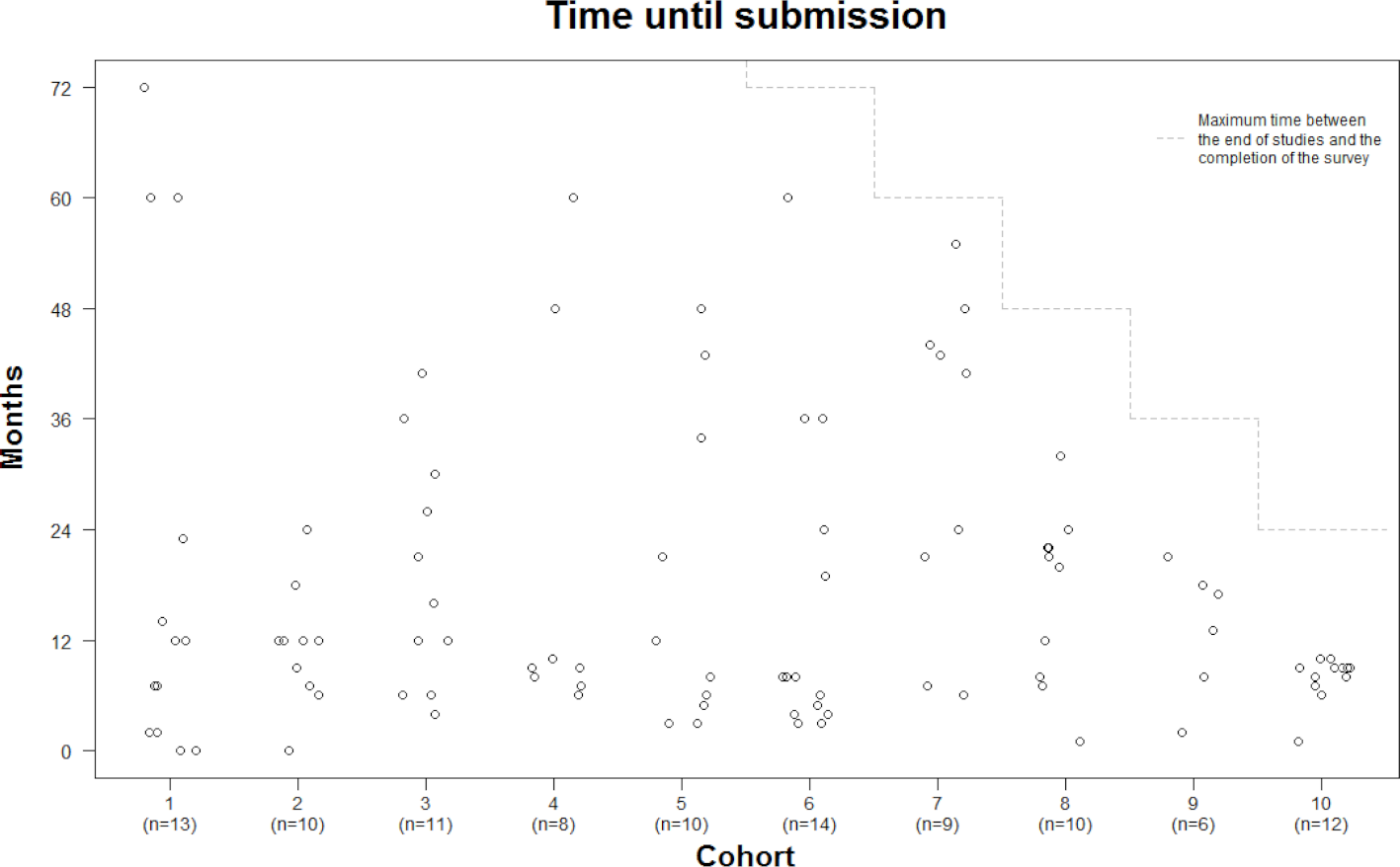
Distribution across the individual cohorts of the time until submission of the master's thesis following the end of studies. As a “censoring effect” might limit information on the later cohorts, we also display the maximum time between the respective end of study and completion of the survey.

157 of the 246 former students participated in the survey (response rate of 63.8%). At least 11 and at most 20 participants were represented from every class. 109 participants (70.8%) had submitted a master's thesis, whereas 45 participants (29.2%) had not completed their studies by submitting a master's thesis (no response for 3 participants). Submission of the master's thesis was equated with a successful completion of studies; only 4 of 246 participants were eliminated during the module phase. Figure 1 (Fig. 1) displays the distribution of the individual submissions of master thesis over time and cohorts. The shortest period of time was the immediate submission at the end of studies and the longest period of time – so far – was 72 months, i.e. six years. All in all, no clear pattern can be discerned, indicating the need for analysing further influencing factors.

#### 3.2 Sociodemographic factors and internal validity

100 men (64.5%) and 55 women (35.5%) took part in the survey (see table 1 (Tab. 1); no response for 2 participants). Participants were between 27 and 61 years old at the start of their studies; the average age was 42.2 years. The comparative data for the total cohorts 1-10 (n=246) were similar (see table 1 (Tab. 1)). 104 out of 151 participants (68.8%) were married, 15.9% lived with partners (n=24), and 15.3% were single, divorced or widowed (n=23). In our analysis, we found no significant effects for the sociodemographic factors and the submission of the master's thesis (see table 2 (Tab. 2)).

#### 3.3 Individual factors with regard to completion of the master's thesis

With respect to the highest academic degree obtained at the start of studies, there was, at 53.3%, a majority of participants with a doctorate (81 of 152) and 36.2% with the with additional habilitation (post-doctoral lecturing qualification; n=55). In the case of 73.2%, advanced studies of the participants were in medicine (112 of 153), whereas dentists (n=17), at 11.1%, and other fields of study were less frequently represented. 79.5% of the participants were working in a clinical specialization (89 of 112). 

75.7% of the participants had turned in the project work (115 of 152). The comparative data for the total cohorts 1-10 (n=246) displayed similar results. Of the 108 participants who completed the master's thesis, all but two participants had turned in all eight follow-up assignments. By contrast, of the 49 participants who did not complete the master's thesis, in 26 cases at least one follow-up assignment was missing. 

The large majority (66.4%) of the participants responded that the choice of topic derived from an idea of their own (95 of 143). With respect to the degree of difficulty, participants responded that in almost half of the cases (48.3%), the choice of topic was made easily or rather easily (72 of 149). For 32.8% of the participants, the choice of topic was difficult or rather difficult (n=49). 

More than half of the participants (63.7%) retained the originally chosen topic (86 of 135). 26 participants (19.2%) changed the topic once; 23 participants (17.0%) changed topic several times. With respect to the study design used, it was shown that in 72.1% of cases (98 of 136), the study design was conceived in the sense of a “clarification” (“Why or how did it work?”; [23]) or “justification study” (“Did it work?”; [23]), whereas purely descriptive studies (“What was done?"; [23]), at 27.9% (n=38), were less frequently represented. 55 of 157 participants (35.0%) indicated that they completed their master's thesis in the period of time originally planned. 53 participants (33.8%) had published their master’s thesis. 

50 published master's theses could be subjected to a more detailed evaluation. In the case of 3 participants, the publication details were not sufficiently comprehensible. 60% of the publications (n=30) took place in specifically education-related journals (GMS Journal for Medical Education (n=15), BMC Medical Education (n=4), European Journal of Dental Education (n=4), Medical Teacher (n=3), Advances in Health Sciences Education (n=1), Advances in Physiology Education (n=1), Medical Education (n=1), Patient Education and Counseling (n=1). In half of the cases (n=15), publication took place in a journal with an impact factor. The latter was between 0.96 and 3.18 and 0.81 on average (including publications with no impact factor). 20 participants (40%), on the other hand, published in a journal of their own specialization (e.g., Anesthesiology, Annals of Anatomy, Journal of Surgical Research, Resuscitation) or thematically-related interdisciplinary journals (The Journal of Medical Internet Research). In these cases, publication only took place in journals with an impact factor. This factor was between 0.14 and 5.12 and 1.93 on average.

The time-to-event analysis (Table 2) showed that participants with a higher academic degree (doctorate or Habilitation) turned in a master’s thesis more frequently than participants with a first degree (master or diploma) or who have passed a state examination. A strong association was observed between turning in the project work and submitting the master's thesis. Further influence could be observed with respect to the issue of topic choice. Participants who had little difficulty in choosing a topic or were not in need for a change of topic had more frequently submitted their master’s thesis. With respect to study design, a “clarification” or “justification study” was more frequently represented among participants who completed their master's thesis as compared to a “descriptive study.”

#### 3.4 External factors with regard to completion of the master's thesis 

With respect to support for the master's thesis by the home faculty (see table 1 (Tab. 1)), it was shown that in the case of granting temporal resources, more than half of the participants (55.6%) found the support to be rather poor or completely inadequate (79 of 142). In the case of technical and substantive support, the share of rather limited or absent support was at 44.8% (66 out of 144). 19.7% of the participants received support from a research assistant, 17.8% from research staff, and 6.4% from a secretary (multiple responses possible). 10.2% of the participants were supported with external funding and 51.0% with office space and materials. There was a significant association between the provision of temporal resources, as well as staff and financial means, and the successful submission of the master's thesis (see table 2 (Tab. 2)). It was also striking that 63.1% of the participants received no financial support (94 of 149) and 64.4% of the participants received no support staff (n=96).

#### 3.5 Program-immanent factors with regard to completion of the master's thesis 

With respect to support for the master's thesis by the program administration (see table 1 (Tab. 1)), it was apparent that 48.9% of the participants regarded the support for finding a topic as complete or adequate (68 of 139). The share of absent or rather inadequate support came to 28.8% (n=40). In the case of the choice of supervisor, the share of completely or rather adequate support was 45.8% (61 of 133) and the share of absent or rather inadequate support was 33.1% (n=44). 42.1% of the participants assessed the significance of the program administration's general support in the modules as completely or rather helpful (59 out of 140), whereas 27.8% judged this aspect to be unhelpful or rather unhelpful (n=39). In the case of over two-thirds of the students (74.1%), the choice of the actual supervisor occurred by way of personal contacts (n=106). More rarely in 16.1%, the choice was made on the basis of recommendations: for example, by the program administration (n=23). 34.5% of the participants (46 of 133) were able to develop a realistic plan of work and schedule with the supervisor, whereas 42.8% of the participants (n=57) did not develop any plan. Half of the participants (68 of 136) early on planned a scholarly publication, whereas in 32.3% of the cases, there was no such planning (n=44). On the participants’ view, the supervisor provided timely feedback in 58.3% (77 of 132) of the cases; in 25.0% of all cases, this was inadequate or largely inadequate (n=33). 62.1% of the participants found the substantive support of the supervisor to be completely or rather adequate (82 of 132); 25 participants (19.0%) had a critical assessment of it. 126 of 134 participants (94.1%) found the supervisor to be competent in the field; eight participants (5.9%) found the competence of the supervisor to be inadequate or largely inadequate. A significant association with submitting the master's thesis (see table 2 (Tab. 2)) could be observed in the case of the general support by the program administration in the modules and in that of ensuring timely feedback. Weak effects were found for a realistic plan of work as well as an early planning of a publication.

#### 3.6 Further influencing factors with regard to completion of the master's thesis 

**Figure 2:**
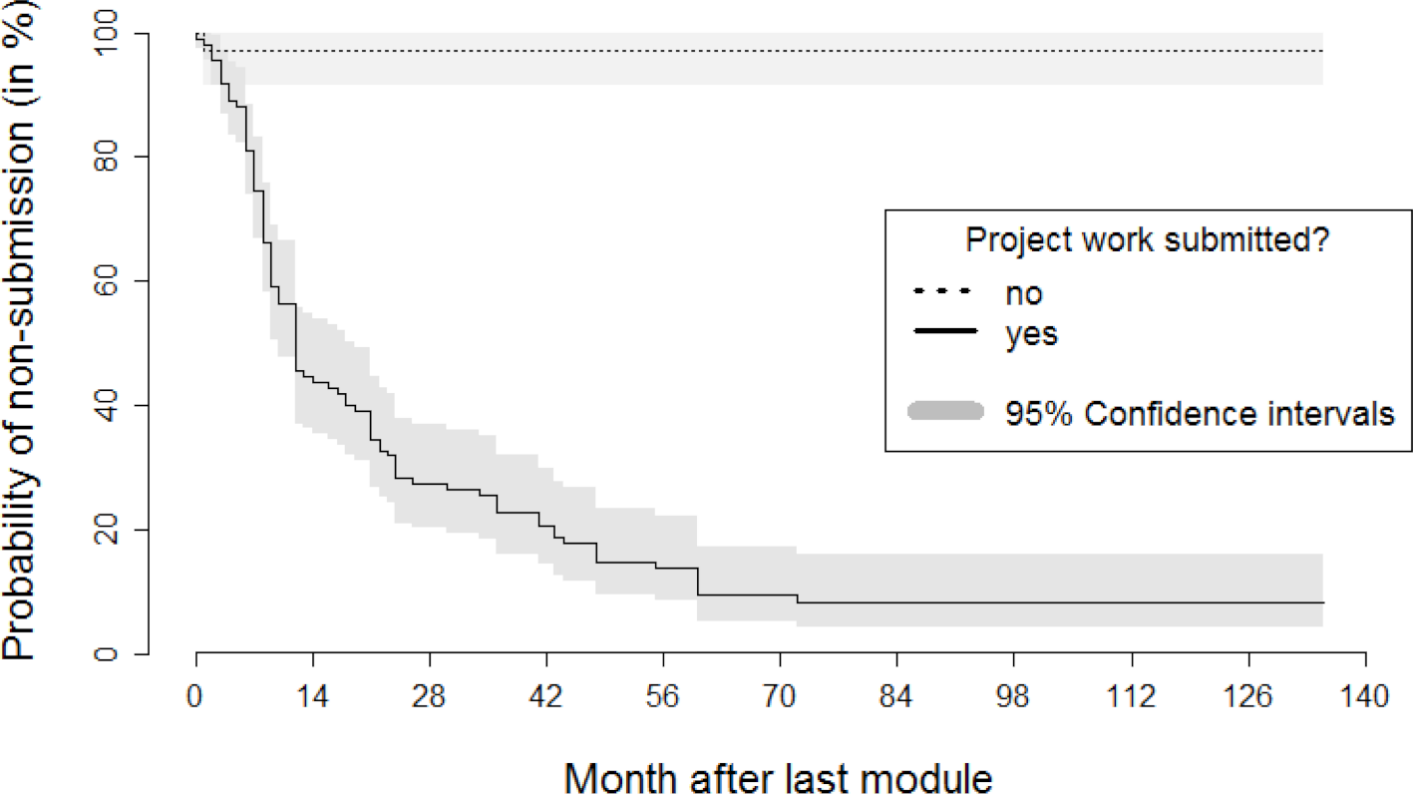
Kaplan-Meier curve on the relationship between turning in the project work and the probability of not turning in the master’s thesis over the course of time after the last module

With respect to further influencing factors (see table 1 (Tab. 1)), only five participants (3.2%) indicated that they had reduced their regular working time due to the study program. More than one-third of the participants (39.4%) completely or largely agreed (52 of 132) that work on their master’s thesis was hindered by non-teaching-related research activity. For 16.2% (21 of 130), this proportion was much lower in the case of teaching-related research projects. By contrast, for almost two-thirds (62.6%), the hindrance caused by other professional projects was much higher (89 of 142). In the case of the factor “hindrance due to a change in professional activity”, 19.6% of the participants (25 of 127) completely or largely agreed. In the case of the factor “hindrance due to one's own illness or the illness of close relations”, at 9.2%, the share of complete or large agreement was small (12 of 130). The factor “hindrance due to parenthood” proved to be more significant: here the share of complete or large agreement was 19.3% (25 of 129). In the case of over one-third of all participants (37.4%), a long-term interruption (>1 year) of work on the master's thesis occurred (52 of 139). A strong association between failure to submit the master's thesis and a long-term interruption of work on the master's thesis was noticed (see figure 2 (Fig. 2)). In addition, hindrances due to other professional projects and a change of professional activity, as well as delays caused by parenthood, exhibit a negative impact on the submission of the master’s thesis. The inhibiting influence is also significant for both teaching-related and non-teaching-related research activity. 

#### 3.7 Development over time

**Figure 3:**
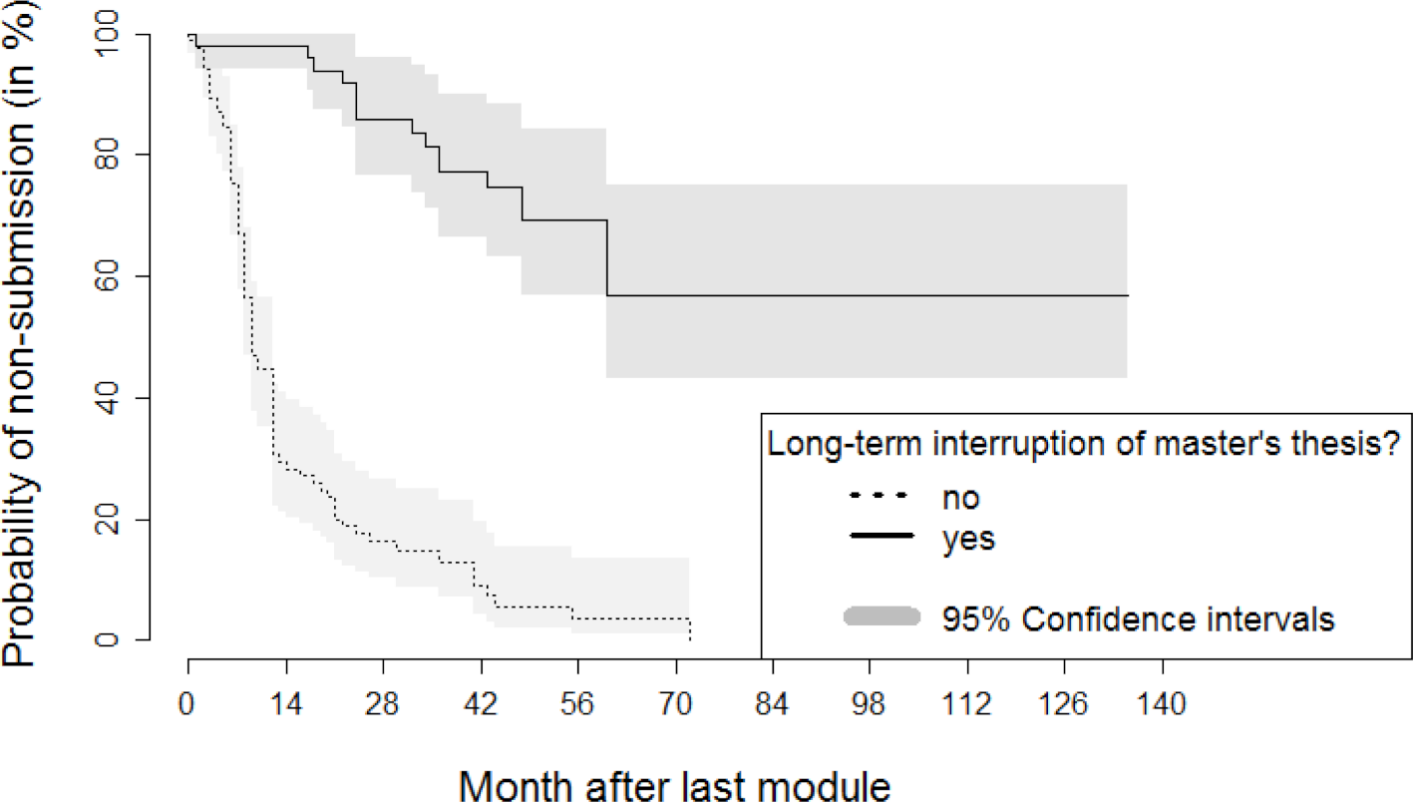
Kaplan-Meier curve on the relationship between the long-term interruption of work on the master's thesis and the probability of not turning in the master's thesis over the course of time after the last module

The previous sections discussed the descriptive findings (see table 1 (Tab. 1)) alongside the time-to-event analysis results of log-rank tests (see table 2 (Tab. 2)). The later assessments on differences in submission rates over the observation period among groups can be visualized very well using Kaplan-Meier curves [19]. For reasons of conciseness, we restrict the following illustration to two of the most influential aspects: the submission of the project work (positive influencing factor) and a long-term interruption (negative influencing factor). Figure 2 (Fig. 2) and figure 3 (Fig. 3) emphasize clearly the according associations in case of both a failure to turn in the project work (see figure 2 (Fig. 2)) or a longer-term interruption of work on the master’s thesis (see figure 3 (Fig. 3)).

## 4. Discussion

Presently, there are only a few studies on the outcome of postgraduate programs in medical education available [8], [24], [25]. An informative analysis and assessment of such programs of study is necessary, in order to evaluate their significance for the professional development of the participants [26] and to be able to respond to current developments with appropriate modifications [13], [27]. In the present survey on the outcome of the MME Germany program, it was possible to achieve a participation rate of 63.8%, which is higher than in comparable studies [c.f. [24]]. The comparative data for the total cohorts 1-10 with regards to sex, age, specialization, and highest academic degree obtained at the start of studies was highly consistent with the data for the survey participants. The distribution of the participants from the individual cohorts displayed a largely equal distribution. Of the 157 participants, 69.4% submitted a master's thesis; the submission rate was thus roughly similar to the completion rates for the first four classes. It can thus be assumed that the field of participants is representative for the program. Significant differences were observed for the time-point of submission of the master's thesis. Thus the question arises which factors influence the outcome criterion of the completion rates and to what extent. 

With respect to the sociodemographic factors, no significant correlations to the submission of the master’s thesis could be observed. With respect to the individual factors, significant differences were found in the case of the highest academic degree obtained as well as turning in the project work. It should be kept in mind here that the project workwas supposed to be prepared during the intensive phase of the program. This suggests that the deficiencies indicate an excessive burden or insufficient structuring and self-organization of the participants [13], [14]. If these traits are only developed to a limited degree among participants in medical education programs that are pursued in parallel with one's profession, the successful completion of the master thesis can represent a substantial challenge. Considerably inhibiting situations can arise especially when unfavourable external factors (e.g., insufficient support at the home faculty) or other factors (e.g., major professional or private life events) come into play at the same time. In order to avoid a failure to meet this challenge, the structuring of one's own work tasks or insight into a greater need for support, and the implementation of the latter, are urgently required [13], [14]. Little difficulty in choosing the topic of the master's thesis and the selection of the study design likewise displayed significant correlations with a successful completion of studies. This could indicate that participants who make a realistic choice of topic also tend to be able to implement a realistic project plan and submission of the thesis. By contrast, a change of topic had a significantly adverse impact on the probability of turning in the thesis. This highlights that already the choice of topic and study design should be made with care and in light of a critical assessment of feasibility, in order to avoid a later change of topic – which is also one of the most important criteria for abandoning studies in the case of medical doctorates [28]. Ideally, the conception of the master's thesis is already combined with planning for a publication and possibly even with the acquisition of external funding. After all, around 10% of the participants succeeded in combining the master's thesis with the acquisition of external funding. Students should also avail themselves of early advising by experienced researchers in education, since, despite many years of professional and publishing activity at the start of studies, they often have only limited experience in the field of educational research. Some of the participants already took advantage of this opportunity, as expressed in the fact that at least 31.8% of the master's theses were published in peer-reviewed journals. The majority of the publications took place in specifically education-related journals. But several participants (12.7%) also displayed their disciplinary ties by publishing in journals in their own field of specialization, which, on the average, have higher impact-factors than the education journals. This made clear that publication of the master's theses is possible in both specifically education-related journals and in field-specific journals. Students may also use the publication for their career ambitions.

With respect to the external factors, serious deficiencies in the participant's perceived support by their home faculties is apparent in the descriptive treatment. Both in the provision of temporal resources and in the granting of staff, funding, materials and office space, the share of inadequate or even completely non-existent support was around 50% or higher. In this connection, the failure to grant temporal resources and support in the form of staff and funding also displayed a significant association to the submission of the master's thesis. The few existing publications on the outcome of medical education programs also mention insufficient time, due to a combination of work and study, and conditions at the home institutions as the most important reasons for not completing one's studies [13]. With regard to these external circumstances, it should be noted that at German faculties of medicine, the aspects of medical pedagogy and educational research only have a short tradition up to now and that until just a few years ago, Germany had a considerable need to catch-up with other countries [29], [30]. The survey participants largely did their studies in this period, such that the survey results reflect the situation at the time. Furthermore, while the number of education-related peer-reviewed journals is constantly increasing, the impact factors for education journals are, for the most part, low. This often makes the publication of the master's theses in disciplinary journals more attractive. In recent years, however, considerable progress could be made in Germany. This gets expressed, among other things, in the fact that university teaching methods courses have become an integral part of concepts of continuing education and advanced training, as well as the fact that professorships and institutions for medical education research and disciplinary teaching methods have been established at several universities [1]. Progress was also apparent in publishing activity [29], [31], and in 2015, “National Competence Based Catalogues of Learning Objectives” for medicine (NKML) and dentistry (NKLZ) could be released in Germany [32]. In light of this background, it can be expected that German home faculties will, in the future, accord even greater importance to medical teaching methods and educational research. The result should be greater support for the program participants on the part of the faculties. 

In considering the program-immanent characteristics, the demands, the organization and the conception of the program have to be taken into account. The program administration initially placed the emphasis on the academic freedom of participants and the scientific rigour of the program [6]. In combination with the lacking prerequisites for implementing the projects, however, it turned out that research-intensive topics often could not be completed, and a change of topic was necessary. At present, only individual reports on completion rates are available, not, however, systematic investigations. Thus, in Maastricht (The Netherlands) the completion rate after more than four years is supposed to be at 93% for at least the first classes [13]. Considering the presently substantial differences in the organization, structure and core contents of medical education programs [8], [9], [10], [11], it must be assumed that up to now neither the level of requirements nor the completion rates of such programs are comparable or, if they are, then only to a limited extent [33]. Moreover, the criteria for the master's thesis in different programs also exhibit significant differences. When the program was accredited in 2012, it was recommended that efforts should be made to increase completion rates in the future [27]. Since a change in the high level of scientific requirements was not compatible with the basic conception of the program, a modification of the original concept occurred in the program, taking into account participant evaluations. This was combined with greater support from the program administration in the choice of topic and more intensive advising. It included the establishment of a separate, additional start module, clarifying the level of requirements, a master's thesis exchange that was integrated into the homepage, and the introduction of the option for several students to elaborate a master's thesis together. In addition, the master's thesis can be submitted as a publication. Whether or not these measures in fact have success has to be assessed in the coming years by way of further studies on the outcome of the program, including the completion rates – though in the last three years of the study period, positive developments are already becoming apparent. The change process of the program-immanent factors, in the sense of continual quality improvement, is thus by no means completed. The survey also showed that the master's theses of the participants who graduated were submitted, on the average, 18 months after the end of studies. This period of time is even less than the recommended completion date of two years after the end of studies. It could be helpful to transform the hitherto only recommended character of this time frame into an obligatory component of the program requirements, as has, for example, already occurred in Maastricht (The Netherlands) and Bern (Switzerland). In Great Britain, the temporal frame for completing the master’s theses is only between six and 12 months [8]. 

With respect to the support provided by the supervisor, it was striking that over two-thirds of the participants chose their supervisor via personal contacts, which showed a significant association to the submission of the master's thesis was also apparent. This suggests the already existing experience on the evaluation of adequate advising, which, given the high proportion of participants with doctorates or Habilitation, is entirely understandable. With respect to ensuring timely feedback and the support and competence of the supervisor, a high to very high proportion of the assessments were positive. The few critical assessments indicate, however, that in individual cases, there is still a discrepancy between the competence in the field, which is actually present, and the implementation of the advising. The cause is probably an excessive burden as a result of the simultaneous supervision of too many theses or as a result of other professional demands [28]. It was shown in the analysis that the factor “ensuring timely feedback in advising” is important; this should be more strongly taken into account in the training and selection of the supervisor. The conclusion of an advising agreement can be a valuable instrument for improving the quality of advising [17]; in it, the rights and obligations of both supervisors and students are already laid down from the start. For this reason, concrete specifications concerning the form of advising in medical education master’s theses would also be desirable. Such specifications are already operative in some places [8], [13]. In order to optimize the support by the supervisors of the master’s theses and to ensure the quality of the program, the results of the alumni survey were summarized in a report and sent to the supervisors. 

With respect to the other factors, a long-term interruption of work on the master's thesis is clearly associated with non-submission of the thesis. This fact should be communicated to the students both in the classroom modules and in the advising. An excessive workload as a result of other professional projects, a change in profession and the start of parenthood could be identified as other hindering or delaying factors. 

## 5. Conclusion

In the present study, it was possible to identify a number of influencing factors for a successful completion of the Master of Medical Education Germany postgraduate program. These include a higher academic degree, little difficulty in choosing a topic, and retention of the originally chosen topic and study design. Choice of the supervisor via personal contacts and ensuring timely feedback by the supervisor proved to be further positive factors. It was also shown that the provision of temporal, staff and financial resources by the home faculty favours the completion of studies. The failure to turn in the project work and a lengthy interruption of master's work could be identified as critical parameters. The evaluation of the actual success of additional program-immanent support measures should be accompanied by further studies on the program outcome. These findings can contribute to increasing the graduation rates in medical education graduate programs. It is plausible that these results can also be applied to other post-graduate programs, since the issue of graduation rates is also reported for dentistry and other degree programs [34], [35], [36]. In order to get more insight in the reasons for not completing the thesis, further research could done by using focus groups and the findings of the research at hand as a starting point. Systematic outcomes research contributes, moreover, to quality assurance in medical education programs and should, in the future, take place in a standardized manner in order to facilitate comparisons between programs. 

## Competing interests

The authors declare that they have no competing interests. 

## Figures and Tables

**Table 1:**
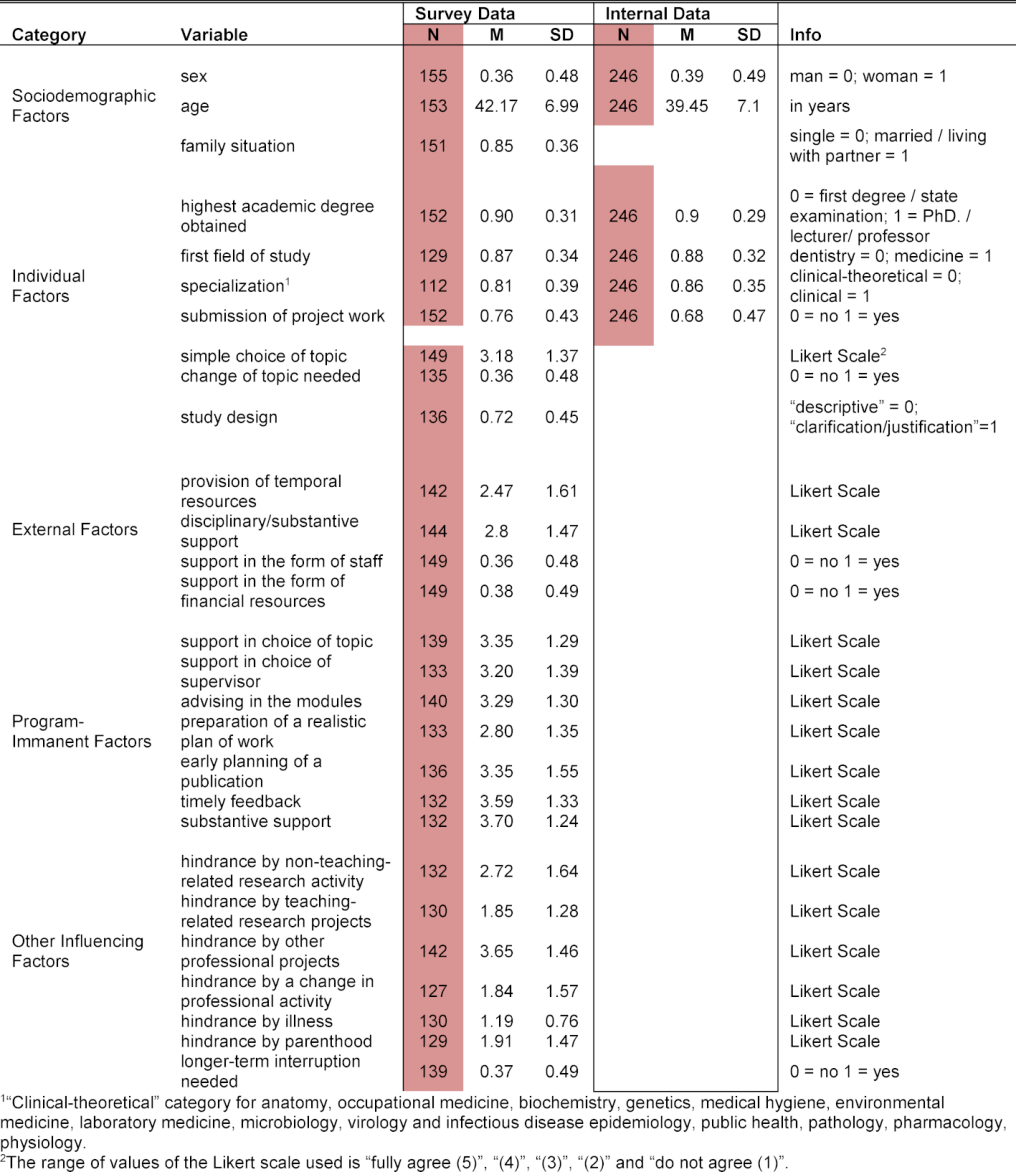
Distribution of sociodemographic, individual, external, program-immanent and other factors influencing the submission of the master's thesis with mean (M) and standard deviation (SD)

**Table 2:**
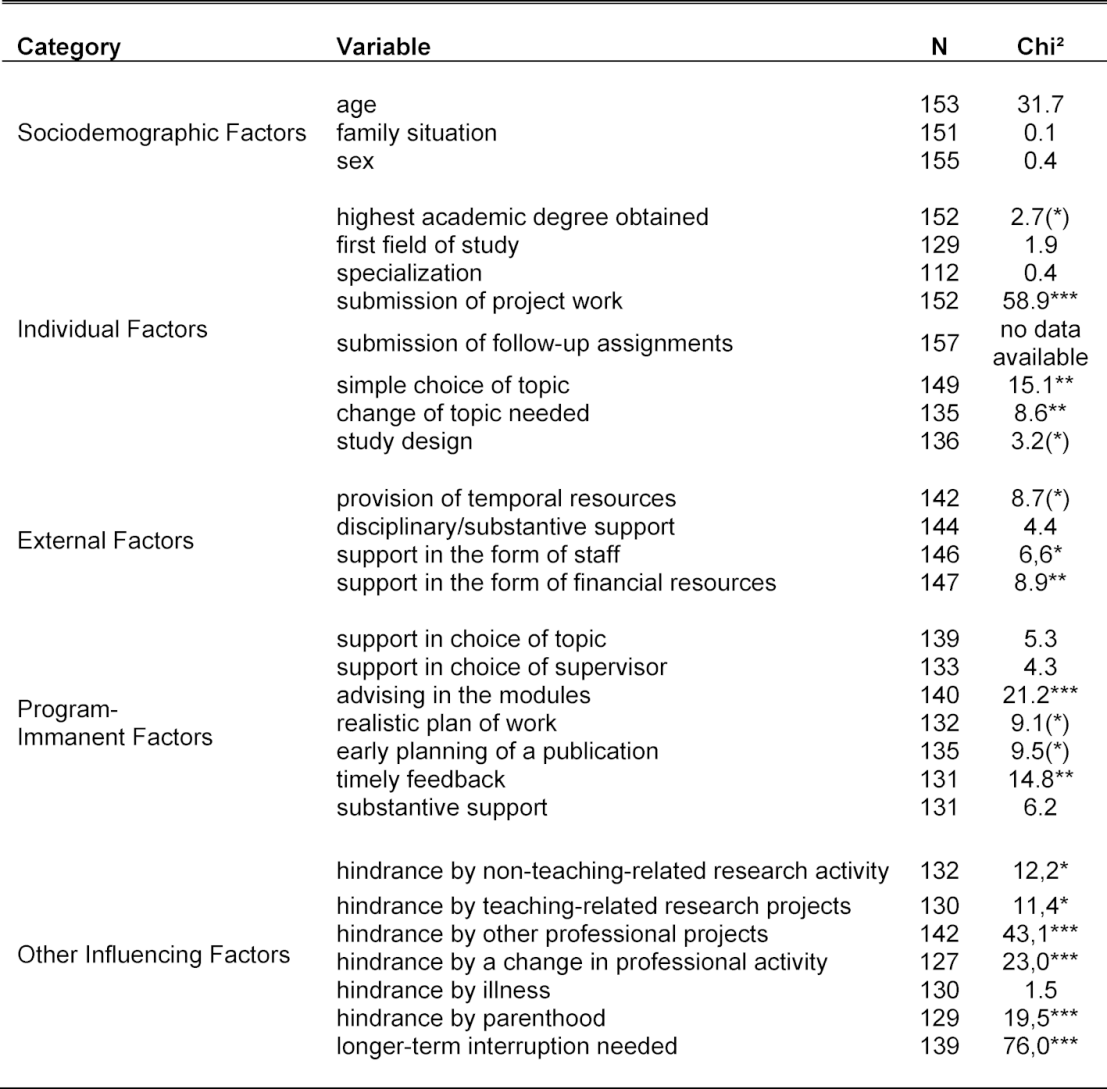
Time-to-event analysis for the submission of the master's thesis and sociodemographic, individual, external, program-immanent and other influencing factors [significance levels determined using log-rank test: 0.001 = ***; 0.01 = ** ; 0.05 = *; 0.1 = (*); N refers to the number of observations as basis for calculation]
